# A Report On Fluctuating Free Convection Flow Of Heat Absorbing Viscoelastic Dusty Fluid Past In A Horizontal Channel With MHD Effect

**DOI:** 10.1038/s41598-020-65252-1

**Published:** 2020-05-22

**Authors:** Farhad Ali, Muhammad Bilal, Madeha Gohar, Ilyas Khan, Nadeem Ahmad Sheikh, Kottakkaran Sooppy Nisar

**Affiliations:** 1 Department of Mathematics, City University of Science and Information Technology, Peshawar, Khyber Pakhtunkhwa Pakistan; 2 Computational Analysis Research Group, Ton Duc Thang University, Ho Chi Minh City, Vietnam; 3 Faculty of Mathematics and Statistics, Ton Duc Thang University, Ho Chi Minh City, Vietnam; 4 Department of Mathematics, College of Science Al-Zulfi, Majmaah University, Al-Majmaah, 11952 Saudi Arabia; 5 Department of Mathematics, College of Arts and Science, Prince Sattam bin Abdulaziz University, Wadi Al-Dawaser, 11991 Saudi Arabia

**Keywords:** Energy science and technology, Nanoscience and technology, Physics

## Abstract

The free convective unsteady fluctuating, MHD flow of electrically conducting viscoelastic dusty fluid in a channel-driven with the impact of oscillating pressure gradient and the motion of the upper plate has been studied in this article. The noteworthy heat generation/absorption has also taken into account, the heat generation established the mechanism of heat transfer by both the momentum of fluid and the motion of dust particle and absorption of heat by the dust particle is because of conduction. The coupled governing partial differential equations are reduced to the ordinary differential equation through the assumed periodic solutions. Analytical solutions for the velocity of the fluid as well as the velocity of dust particles and for energy equation of the fluid and for dust particles are obtained by using Poincare-Light Hill Perturbation Technique. The influence of various parameters of interest is discussed on the velocity and temperature profiles of the fluid and particles. The evolution of fluid-phase and dusty-phase with dual behavior of the magnetic parameter for both boundary layer and free stream velocities has been discussed. The boundary layer velocity decreased with an increase in magnetic parameter, while at the free stream flow, the result is quite opposite. The above result of magnetic field is worthwhile and can be used to control the boundary layer thickness. The current work also concludes that by increasing the Peclet number and concentration of the dust particles retards the boundary layer velocity. Furthermore, various physical parameters like coefficient of heat absorption, concentration of the dust particles, peclet number, magnetic parameter, and temperature relaxation time parameter retard the motion of dusty-phase, while Grashof number enhances the flow of dusty-phase. Other properties of fluid, which have great importance for engineers are, the rate of heat transfer and skin friction. It is shown in Table 1 that by increasing the value of Peclet number from 1 to 2 it increases the rate of heat transfer from 1.3263 to 1.3387. Furthermore, Table 2 shows that by increasing the concentration parameter from 2 to 4 the skin friction increases from 2.3872 to 4.7799.

## Introduction

Magnetohydrodynamic free convection is paramount due to the existence in natural and as well as in fluid engineering problems. Applications of free convection in natural phenomena are: the ocean currents which are generated from the forces acting upon the water like temperature and salinity differences, sea wind formation and the rising plume of hot air is just because of convection. While in the fluid engineering problems the MHD flows with free convection are used in MHD generators, accelerators, flow meters, blood flow, enhancement of heat transfer in gas cooling systems, geothermal energy extraction are the areas having great technical importance in fluid engineering problems. Keeping in view all aforementioned applications of free convective flow with transversely applied magnetic field, many researchers investigated various directions of this phenomenon, like Ahmad *et al*.^1^, Takhar *et al*.^2^, Graham^3^, Gupta^4,5^, Helmy^6^, Hossain^7^, Aldoss *et al*.^8^, Kuiken^9^, and many more worked out the analysis of MHD flow with free convection but in all these investigations the influence of heat generation/absorption by the fluid was not taken into account. While in many real-world industrial applications, like the storage of edible stuff, exothermic/endothermic chemical reactions, the removal of heat from nuclear fuel debris, etc the influence of heat absorption/generation is of great importance. Due to this significance, the unsteady MHD flow of a particular suspension in a conducting fluid flowing in a channel in the presence of thermal radiation and heat generation/absorption is analyzed by Chamkha^10^. Electrically conducting MHD free convection flow of incompressible viscous fluid past over a porous plate with uniform suction and heat transfer is investigated by Sahoo *et al*.^11^. The combined influence of heat generation and chemical reaction on MHD free convection flow over a movable plate embedded in a porous medium is discussed by Khan *et al*.^12^. Furthermore, the heat generation and chemical reaction for generalized Casson fluid model has been discussed by Sheikh *et al*.^13^. They have performed the comparison of Atangana–Baleanu and Caputo– Fabrizio fractional derivatives for the above-mentioned fluid. Furthermore, MHD flows are widely used in microfluidic flows. Microfluidic are those flows that are geometrically constrained to a small capillary penetration which governs mass transport. Qain and Bau^14^ have numerically discussed the two dimensional steady and unsteady flow govern by electric field in cavity. The translational motion of particles in non-uniform converging and diverging microchannel has been analyzed by Zhou *et al*.^15^. For the first time, a finite element model was employed for investigation of conditions for dielectrophoretic (DEP) chocking in a converging-diverging microchannel by Ai *et al*.^16^. They assumed large size particles. The size of the particle is larger as compared to electric double layer in the vicinity of charged particle and the channel wall. So that’s why their results were not applicable for nano-particles for which the hydrodynamic and electrokinetics in DEL must be fully resolved. Furthermore Zhou *et al*.^17^ in another paper develops a numerical simulation model to investigate the deformable particle-particle interaction with an impact of dielectrophoresis (DEP) under an electric field induced by alternating current.

Over the past few decades the researchers are working on multi-phase MHD dusty flows due to its great importance in applications of fluidization, flows in tubes of a rocket, flow of blood in arteries, DPDs (dusty plasma devices), MHD generators, accelerators, electrostatic precipitator and the use of dust in gas cooling systems. One can go through the literature and can find different types of multiphase flows, but the most common class of multiphase flows are two-phase flows, which consist of liquid-gas flow, liquid-liquid flow, solid-gas flow, and liquid-solid flows. Soo^18^, for the first time, introduced the basic theory of multi-phase flows. In the same decade number of researchers like Michael and Miller^19^, Saffman^20^, Healy^21^, Vimala^22^, Gupta and Gupta^23^, Venkateshappa *et al*.^24^, Venkatesh and Kumara^25^, Ghosh and Sana^26^, Gosh and Gosh^27^, Gireesha *et al*.^28^, Gosh and Debnath^29^, Attia and Abdeen^30^ and many other researchers have been dealing with theoretical modeling and experimental measurement of particle-phase viscosity in a multi-phase dusty fluid.

Viscosity and elasticity are the properties of liquid and solid respectively. The materials having both the characteristics of viscosity and elasticity are termed as viscoelastic materials. The applications of viscoelastic fluids can be found in many polymer industries and in blood flows. Due to the elastic nature blood store energy in the circulatory system, because of the viscous nature a partial part of the energy is dissipated to heat, while the remaining part of the energy is related to movement^31^, so keeping in mind this behavior of blood viscoelasticity is extremely important in blood flows. Relaxation time and retardation time are the two important properties of viscoelastic fluids. For the case when both the properties are zero, the fluid is called Newtonian. When relaxation is zero and retardation is positive then such fluid is second-grade fluid, relaxation is zero and retardation is negative the fluid will be Walter’s B fluid and the fluid will be Maxwell when relaxation is non-zero and retardation is zero. For characterizing the creeping nature of viscoelastic fluid Oldroyd^32^ has modeled the constitutive equations, and also investigated the influence of non-Newtonian fluid flow using the Oldroyd fluid model. Rajagopal and Bhatnagar^33^ derived the analytical solutions of some flows governed by Oldroyd fluid model. Ali *et al*.^34^ worked out the influence of various physical parameters on the fluctuating flow of viscoelastic dusty fluid flows in a channel with heat transfer. The increase of retardation time increases the elastic nature of the fluid which causes a decrease in fluid velocity, Ali *et al*.^35^ investigated the second-grade viscoelastic fluid with free convection from their findings they reflect the same results that increase of second-grade parameter causes retardation in fluid velocity. The flow of the viscoelastic solution is worked out by Zhou *et al*.^36^. They considered that the particle flow is driven by dielectrophoretic (DEP) forces induced by applied electric field.

In the above literature, the researchers have investigated free convection MHD flow of Newtonian, incompressible, electrically conducting dusty fluids. To the best of our knowledge, no study has been reported to couple the energy equation of the fluid to the energy equation of the dust particle for non-Newtonian viscoelastic fluid. By considering the separate energy equation for the dust particle is quite challengeable, especially to investigate the analytical solutions. Therefore in the present work, we have tried to theoretically investigate free convection fluctuating MHD flow of viscoelastic fluid having suspended conducting particles with heat generation in a horizontal channel driven by an oscillating pressure gradient, along with a separate heat equation for the conducting dust particles. Furthermore, this work unexplored the effect of various physical parameters and heat absorption on the dusty flow of non-Newtonian viscoelastic fluid.

## Mathematical Formulation

The fully developed free stream fluctuating, incompressible, the unidirectional unsteady flow of electrically conducting viscoelastic dusty fluid in a channel has been considered in this study. The magnetic field has applied transversely to the flow of viscoelastic dusty fluid. Due to the small magnetic Renolds number i.e Re_*m*_ ≪ 1 the induced magnetic field and the electric field due to the polarization of charges are ignored. Flow generation is caused by the application of oscillating pressure gradient, the motion of the upper plate with free stream velocity *U*(*t*) which is independent of space variable and heat transfer. One-dimensional flow has been considered along *x*-axis between two parallel plates. In the energy equation radiation and viscous dissipation have not been considered.

**Figure 1:**
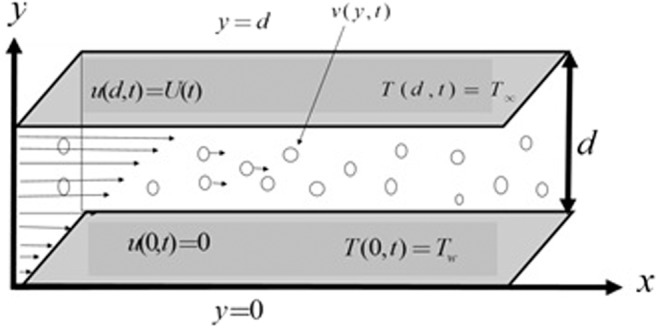
Schematic diagram of the flow.

The lower plate has zero velocity while the upper plate is oscillating with free stream velocity *U*(*t*). $u(y,t),\,v(y,t)$ show the velocity of fluid and velocity of dust particles. The lower plate having ambient temperature *T*_∞_ while T_*w*_ is the temperature of the upper plate, and *T*_*p*_ represents the particles’ temperature as shown in Fig. 1. To avoid likeness it is preferred to refs. ^37–40^ for momentum and energy equations of fluid and dust particles.

1 $$\frac{\partial u}{\partial t}=-\frac{1}{\rho }\frac{\partial p}{\partial x}+\left(\upsilon +\frac{{\alpha }_{1}}{\rho }\frac{\partial }{\partial t}\right)\frac{{\partial }^{2}u}{\partial {y}^{2}}+\frac{{K}_{0}{N}_{0}}{\rho }(v-u)-\frac{\sigma {B}_{0}^{2}u}{\rho }+g{\beta }_{T}(T-{T}_{\infty }),$$

2 $$m\frac{\partial v}{\partial t}={K}_{0}(u-v),$$

3 $$\frac{\partial T}{\partial t}=\frac{k}{\rho {c}_{p}}\frac{{\partial }^{2}T}{\partial {y}^{2}}+\frac{{\rho }_{p}{c}_{s}}{\rho {c}_{p}{\gamma }_{T}}({T}_{p}-T)-\frac{{Q}_{0}T}{\rho {c}_{p}},$$

4 $$\frac{\partial {T}_{p}}{\partial t}=\frac{1}{{\gamma }_{T}}(T-{T}_{p}).$$

The concern boundary conditions are:

5 $$\begin{array}{c}u(y,0)=0,\,T(y,0)={T}_{\infty ,}\,t\le 0,\\ u(0,t)=0,\,T(0,t)={T}_{w,}\,y=0,\,t > 0,\,\\ u(d,t)=U(t),\,T(d,t)={T}_{\infty ,}\,y=d,t > 0,\end{array}$$

to find the velocity of the dust particle let assume that $v(y,t)={v}_{0}(y){e}^{i\omega t},$ by incorporating this value of $v(y,t)$ in Eq. (2) velocity of the dust particles will be:

6 $$v(y,t)=(\frac{{K}_{0}}{mi\omega +{K}_{0}})u(y,t),$$

using Eq. (6) in Eq. (1) the momentum equation will be:

7 $$\frac{\partial u}{\partial t}=-\frac{1}{\rho }\frac{\partial p}{\partial x}+\left(\upsilon +\frac{{\alpha }_{1}}{\rho }\frac{\partial }{\partial t}\right)\frac{{\partial }^{2}u}{\partial {y}^{2}}+\frac{{K}_{0}{N}_{0}\,u}{\rho }\left\{\left(\frac{{K}_{0}}{mi\omega +{K}_{0}}\right)-1\right\}-\frac{\sigma {B}_{0}^{2}u}{\rho }+g{\beta }_{T}(T-{T}_{\infty }).$$

At free stream area Eq. (7) will adopt the following form:

8 $$\frac{\partial u}{\partial t}=\frac{dU}{dt}+\upsilon \frac{{\partial }^{2}u}{\partial {y}^{2}}+\frac{{\alpha }_{1}}{\rho }\frac{{\partial }^{3}u}{\partial t\partial {y}^{2}}-\frac{\sigma {B}_{0}^{2}}{\rho }(u-U)+\frac{{K}_{0}{N}_{0}}{\rho }\left(\frac{{K}_{0}}{(mi\omega +{K}_{0})}-1\right)(u-U)+g{\beta }_{T}(T-{T}_{\infty }).$$

### Solution of the problem

For the solutions of the coupled partial differential equation, The Poincare-Lighthill Perturbation Technique^41^ has been employed. For non-dimensionalization of Eqs. (3, 4 and 8) introducing the following dimensionless variables.

9 $$\begin{array}{c}{u}^{\ast }=\frac{U}{{U}_{0}},\,{y}^{\ast }=\frac{y}{d},\,{t}^{\ast }=\frac{{U}_{0}t}{d},\\ \theta =\frac{T-{T}_{\infty }}{{T}_{w}-{T}_{\infty }},\,{\theta }_{p}=\frac{{T}_{p}-{T}_{\infty }}{{T}_{w}-{T}_{\infty }},\,{\tau }^{\ast }=\frac{\tau {d}^{2}}{\mu \upsilon },\end{array}$$

dimensionless governing equations for the momentum of the fluid, energy of the fluid and energy of the dust particles are given as:

10 $$\mathrm{Re}\frac{\partial u}{\partial t}=\mathrm{Re}\frac{dU}{dt}+\frac{{\partial }^{2}u}{\partial {y}^{2}}+\alpha \frac{{\partial }^{3}u}{\partial t\partial {y}^{2}}+({K}_{2}-{K}_{1})(U-u)-M(u-U)+Gr\theta ,$$

11 $$\frac{\partial \theta }{\partial t}=\frac{1}{Pe}\frac{{\partial }^{2}\theta }{\partial {y}^{2}}+\frac{R}{Pe}({\theta }_{p}-\theta )-\phi \theta ,$$

12 $$\frac{\partial {\theta }_{p}}{\partial t}=\gamma (\theta -{\theta }_{p}),$$

with dimensionless boundary conditions,

13 $$\begin{array}{c}u(0,t)=0,\,u(1,t)=U(t),\,\theta (0,t)=1,\,\theta (1,t)=0,\\ U(t)=1+\frac{{\boldsymbol{\varepsilon }}}{2}({e}^{i\omega t}+{e}^{-i\omega t}).\end{array}$$

during the calculi, the following physical parameters appear:

14 $$\begin{array}{c}\mathrm{Re}=\frac{{u}_{0}d}{\upsilon },\,\alpha =\frac{{\alpha }_{1}{u}_{0}}{\rho \upsilon d},\,{K}_{1}=\frac{{K}_{0}{N}_{0}{d}^{2}}{\rho \upsilon },\,{K}_{2}=\frac{{K}_{0}^{2}{N}_{0}{d}^{2}}{\rho \upsilon (mi\omega +{K}_{0})},\\ M=\frac{\sigma {B}_{0}^{2}{d}^{2}}{\rho \upsilon },\,Gr=\frac{g{\beta }_{T}{d}^{2}({T}_{w}-{T}_{\infty })}{\upsilon {u}_{0}},\,Pe=\frac{\rho {c}_{p}{u}_{0}d}{k},\\ \,\frac{1}{Pe}=\frac{k}{\rho {c}_{p}{U}_{0}d},\,R=\frac{{d}^{2}{\rho }_{p}{c}_{p}}{{\gamma }_{T}K},\,\phi =\frac{d{Q}_{0}}{\rho {c}_{p}{U}_{0}},\end{array}$$

to find ${\theta }_{p}$, let assume that ${\theta }_{p}(y,t)={\theta }_{p}(y){e}^{i\omega t},$ by incorporating these values in Eq. (12) the value of ${\theta }_{p}$ will be

15 $${\theta }_{p}(y,t)=\left(\frac{\gamma }{i\omega +\gamma }\right)\theta .$$

Using Eq. (15) in Eq. (11) the energy equation becomes:

16 $$\frac{\partial \theta }{\partial t}=\frac{1}{Pe}\frac{{\partial }^{2}\theta }{\partial {y}^{2}}+\frac{R}{Pe}\left(\frac{\gamma \,\theta }{i\omega +\gamma }-\theta \right)-\phi \theta .$$

to solve energy equation let assume the periodic solution of the form

17 $$\,\theta (y,t)={\theta }_{0}(y)+\varepsilon {\theta }_{1}(y){e}^{i\omega t}.$$

Using this assumption and dimensionless boundary condition from Eq. (13) in Eq. (16), the non-harmonic and harmonic parts of the energy equation are given as

18 $$\,{\theta }_{0}(y)=\frac{\sinh \,[\sqrt{{m}_{0}}-\sqrt{{m}_{0}}y]}{\sinh \,[\sqrt{{m}_{0}}]},\,{\theta }_{1}(y)=0.$$

Using Eq. (15) in Eq. (14) the solution of the energy equation will be

19 $$\theta (y,t)=\left(\frac{\sinh \,[\sqrt{{m}_{0}}-\sqrt{{m}_{0}}y]}{\sinh \,[\sqrt{{m}_{0}}]}\right).$$

Equation (19) satisfies boundary conditions which show the validity of calculations. By incorporating Eq. (19) in Eq. (10) the momentum equation becomes

20 $$\mathrm{Re}\frac{\partial u}{\partial t}=\mathrm{Re}\frac{dU}{dt}+\frac{{\partial }^{2}u}{\partial {y}^{2}}+\alpha \frac{{\partial }^{3}u}{\partial t\partial {y}^{2}}+({K}_{2}-{K}_{1})(U-u)-M(u-U)+Gr\left(\frac{\sinh \,[\sqrt{{m}_{0}}-\sqrt{{m}_{0}}y]}{\sinh \,[\sqrt{{m}_{0}}]}\right).$$

By assuming the following periodic solution for the momentum of the fluid as:

21 $$u(y,t)={u}_{0}(y)+\frac{\in }{2}({u}_{1}(y)+{u}_{2}(y)){e}^{i\omega t}.$$

using Eq. (13) and Eq. (21) in Eq. (20), the harmonic and non-harmonic systems are:

22 $${u}_{0}(y)=-B\frac{\sinh \,[\sqrt{H}-\sqrt{H}y]}{\sinh \,[\sqrt{H}]}+1+(B-1)\frac{\sinh \,[\sqrt{{m}_{0}}-\sqrt{{m}_{0}}y]}{\sinh \,[\sqrt{{m}_{0}}]}.$$

23 $${u}_{1}(y)=\,\frac{\sinh \,\sqrt{{m}_{2}}y-\sqrt{{m}_{2}}}{\sinh \,\sqrt{{m}_{2}}}+1,$$

24 $$\,{u}_{2}(y)=\frac{\sinh \,[\sqrt{{m}_{3}}\,y-\sqrt{{m}_{3}}]}{\sinh \,[\sqrt{{m}_{3}}]}+1,$$

using Eqs. (22, 23 & 24) in Eq. (21) the final velocity of the fluid will be

25 $$\begin{array}{c}u(y,t)=\,\left(-B\frac{\sinh \,[\sqrt{H}-\sqrt{H}y]}{\sinh \,[\sqrt{H}]}+1+(B-1)\frac{\sinh \,[\sqrt{{m}_{0}}-\sqrt{{m}_{0}}y]}{\sinh \,[\sqrt{{m}_{0}}]}\right)\,\\ +\,\frac{\in }{2}\left[\left(\frac{\sinh \,\sqrt{{m}_{2}}y-\sqrt{{m}_{2}}}{\sinh \,\sqrt{{m}_{2}}}+1\right){e}^{i\omega t}+\left(\frac{\sinh \,[\sqrt{{m}_{3}}\,y-\sqrt{{m}_{3}}]}{\sinh \,[\sqrt{{m}_{3}}]}+1\right){e}^{-i\omega t}\right].\end{array}$$

where

$$\begin{array}{ccc}{m}_{0} & = & \frac{{R}_{1}(\gamma +i\omega )+Pe\gamma \phi -{R}_{1}\gamma -(\gamma +i\omega )\phi }{(\gamma +i\omega )},\\ {m}_{2} & = & \frac{\mathrm{Re}i\omega -H}{1+\alpha },\,{m}_{3}=\frac{\mathrm{Re}i\omega +H}{1-\alpha },\\ H & = & {K}_{1}-{K}_{2}-M,\,B=1+\frac{Gr}{H-{m}_{0}}.\,\end{array}.$$

Equation (25) satisfies the boundary conditions which guarantee the validation of performed calculations.

### Nusselt number

The evaluation of the rate of heat transfer from Eq. (19) is

$$Nu={\frac{\partial \theta }{\partial y}|}_{y=0}=\sqrt{{m}_{0}}\frac{\cosh \,[\sqrt{{m}_{0}}]}{\sinh \,[\sqrt{{m}_{0}}]},$$

### Skin friction

To find the skin friction for viscoelastic dusty fluid, the dimensional form for finding *C*_*f*_ is given as:

26 $$\tau =\mu \frac{\partial u}{\partial y}+{\alpha }_{1}\frac{{\partial }^{2}u}{\partial t\partial y}.$$

To make Eq. (26) dimensionless using the dimensionless variable from (9) we will get the following dimensionless form for skin friction, the (*) sign has been omitted for the sake of simplicity.

27 $$\tau =\mathrm{Re}\frac{\partial u}{\partial y}+\alpha \frac{{\partial }^{2}u}{\partial t\partial y}.$$

**Table 1:** Numerical interpretation of Nusselt number with various physical parameters of interest.

*Pe*	*γ*	*ϕ*	*R*	*ω*	Nu
**1**	1.5	0.1	2	π/2	1.3263
**2**	1.5	0.1	2	π/2	1.3387
1	**0.5**	0.1	2	π/2	1.5224
1	**1**	0.1	2	π/2	1.4261
1	1.5	**0.2**	2	π/2	1.3095
1	1.5	**0.4**	2	π/2	1.2757
1	1.5	0.1	**3**	π/2	1.4917
1	1.5	0.1	**4**	π/2	1.6512

**Table 2:** Numerical interpretation of skin friction with different physical parameters.

*Pe*	*Gr*	*M*	*K*	*α*	*R*	Re	*γ*	*ϕ*	*t*	*ω*	*ε*	*Cf*
**1**	0.7	0.5	0.5	0.5	2	1.5	1.5	1	1	π/6	0.001	2.3927
**2**	0.7	0.5	0.5	0.5	2	1.5	1.5	1	1	π/6	0.001	2.3790
**3**	0.7	0.5	0.5	0.5	2	1.5	1.5	1	1	π/6	0.001	2.3654
1	**1**	0.5	0.5	0.5	2	1.5	1.5	1	1	π/6	0.001	2.5905
1	**1.5**	0.5	0.5	0.5	2	1.5	1.5	1	1	π/6	0.001	2.9202
1	**2**	0.5	0.5	0.5	2	1.5	1.5	1	1	π/6	0.001	3.2500
1	0.7	**1**	0.5	0.5	2	1.5	1.5	1	1	π/6	0.001	2.0594
1	0.7	**2**	0.5	0.5	2	1.5	1.5	1	1	π/6	0.001	1.3140
1	0.7	**3**	0.5	0.5	2	1.5	1.5	1	1	π/6	0.001	0.4294
1	0.7	0.5	**1**	0.5	2	1.5	1.5	1	1	π/6	0.001	2.7045
1	0.7	0.5	**2**	0.5	2	1.5	1.5	1	1	π/6	0.001	3.2748
1	0.7	0.5	**3**	0.5	2	1.5	1.5	1	1	π/6	0.001	4.9140
1	0.7	0.5	0.5	**2**	2	1.5	1.5	1	1	π/6	0.001	3.5892
1	0.7	0.5	0.5	**3**	2	1.5	1.5	1	1	π/6	0.001	3.5872
1	0.7	0.5	0.5	**4**	2	1.5	1.5	1	1	π/6	0.001	3.5855
1	0.7	0.5	0.5	0.5	**4**	1.5	1.5	1	1	π/6	0.001	4.7799
1	0.7	0.5	0.5	0.5	**5**	1.5	1.5	1	1	π/6	0.001	5.9737
1	0.7	0.5	0.5	0.5	**6**	1.5	1.5	1	1	π/6	0.001	7.1673
1	0.7	0.5	0.5	0.5	2	**2**	1.5	1	1	π/6	0.001	2.3910
1	0.7	0.5	0.5	0.5	2	**3**	1.5	1	1	π/6	0.001	3.5857
1	0.7	0.5	0.5	0.5	2	**4**	1.5	1	1	π/6	0.001	4.7799
1	0.7	0.5	0.5	0.5	2	1.5	**3**	1	1	π/6	0.001	1.7943
1	0.7	0.5	0.5	0.5	2	1.5	**4.5**	1	1	π/6	0.001	1.7968
1	0.7	0.5	0.5	0.5	2	1.5	**6**	1	1	π/6	0.001	1.7979
1	0.7	0.5	0.5	0.5	2	1.5	1.5	**2.5**	1	π/6	0.001	1.8057
1	0.7	0.5	0.5	0.5	2	1.5	1.5	**3.5**	1	π/6	0.001	1.8180
1	0.7	0.5	0.5	0.5	2	1.5	1.5	**4.5**	1	π/6	0.001	1.8296
1	0.7	0.5	0.5	0.5	2	1.5	1.5	1	**2**	π/6	0.001	1.7886
1	0.7	0.5	0.5	0.5	2	1.5	1.5	1	**3**	π/6	0.001	1.7922
1	0.7	0.5	0.5	0.5	2	1.5	1.5	1	**4**	π/6	0.001	1.7995
1	0.7	0.5	0.5	0.5	2	1.5	1.5	1	1	**π/6**	0.001	1.8002
1	0.7	0.5	0.5	0.5	2	1.5	1.5	1	1	**π/4**	0.001	1.7967

## Results and discussion

Parametric influence for various physical parameters of our interest on the velocity profile of fluid, velocity profile of particles, skin friction and on the rate of heat transfer have been discussed in the current study. Figures 2, 3, 4, 5, 6 and 7 have been plotted for the influence of different physical parameters on velocity of the fluid, Figs. 8, 9, 10, 11, 12, 13 and 14 have been drawn for parametric influence of velocity profile of the dust particles, variation of skin friction which is the applied shear stresses at *y* = 0 have shown in Figs. 15, 16, 17, 18, 19 and 20.

**Figure 2:**
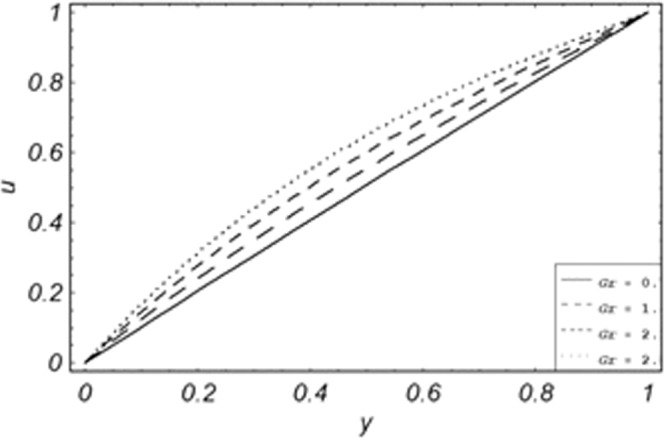
Velocity evolution with different values of *Gr*.

**Figure 3:**
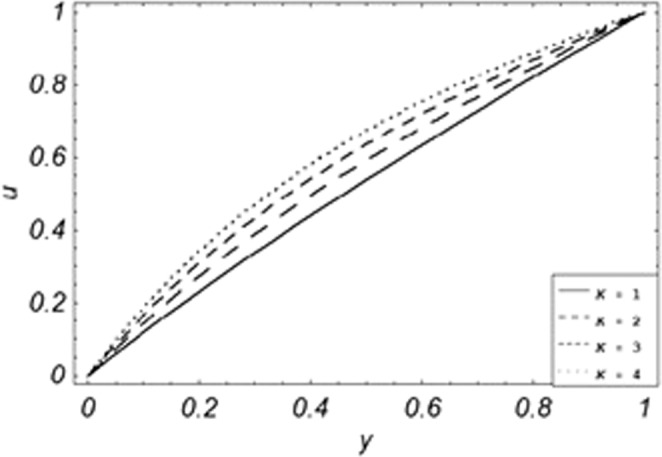
Velocity evolution with different values of *K*.

**Figure 4:**
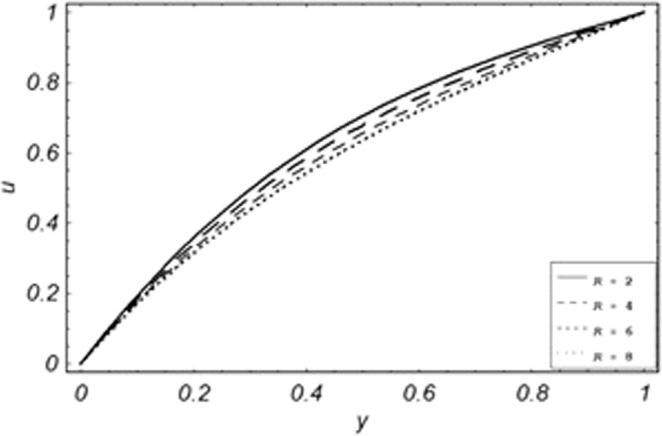
Velocity evolution with different values of *R*.

**Figure 5:**
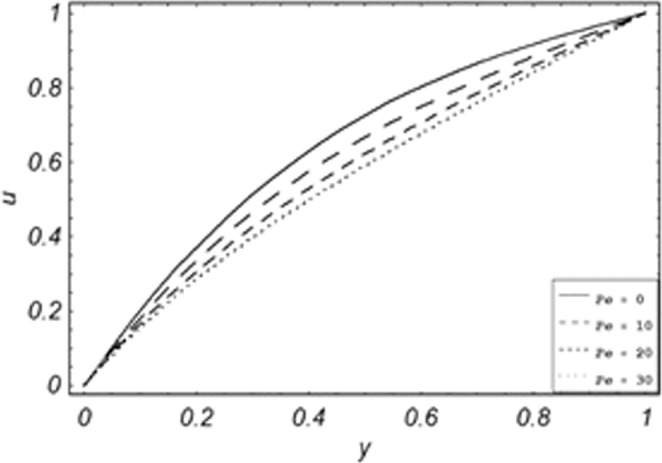
Velocity evolution with different values of *Pe*.

**Figure 6:**
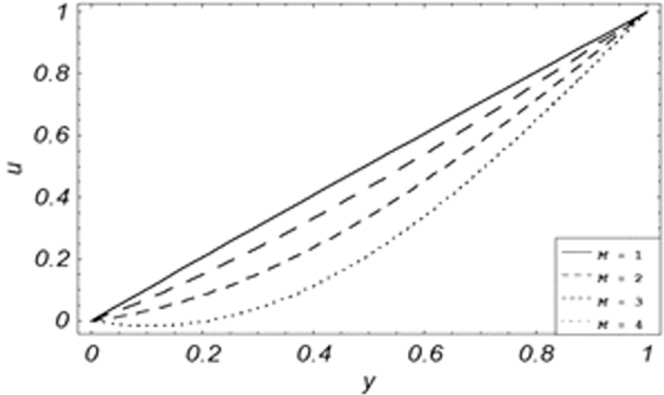
Velocity evolution with different values of *M*.

**Figure 7:**
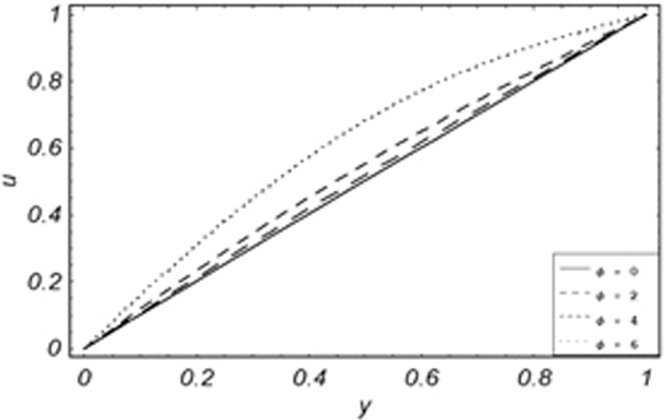
Velocity evolution with different values of *ϕ*.

**Figure 8:**
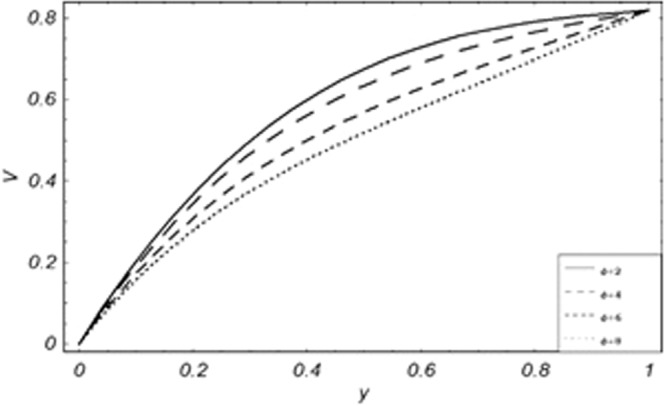
Variation of particle velocity with various values of *ϕ*.

**Figure 9:**
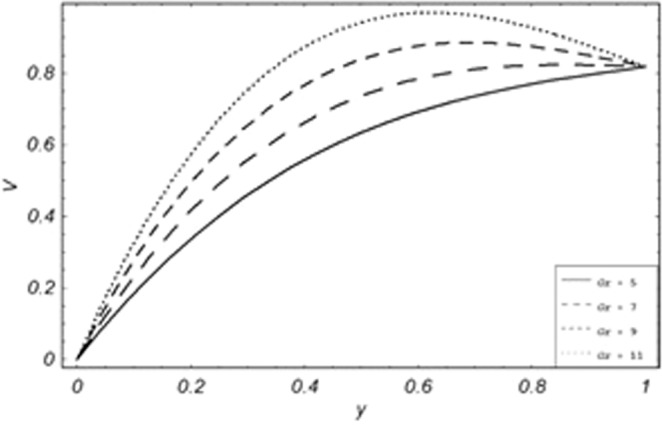
Variation of particle velocity with the various value of *Gr*.

**Figure 10:**
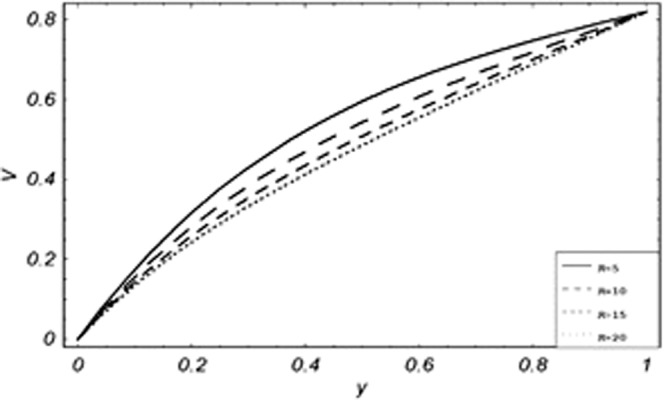
Variation of particle velocity with different values of *R*.

**Figure 11:**
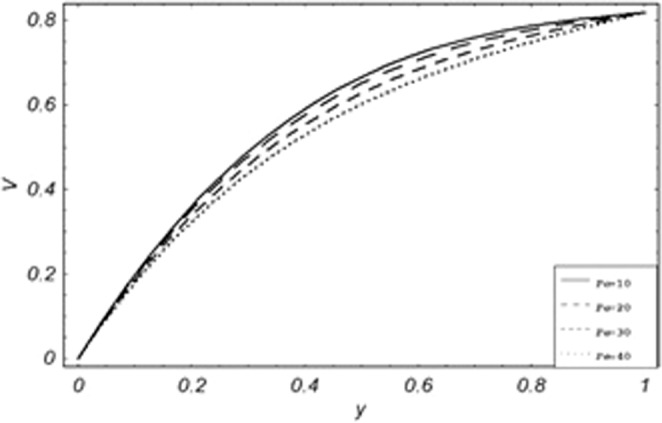
Variation of particle velocity with different values of *Pe*.

**Figure 12:**
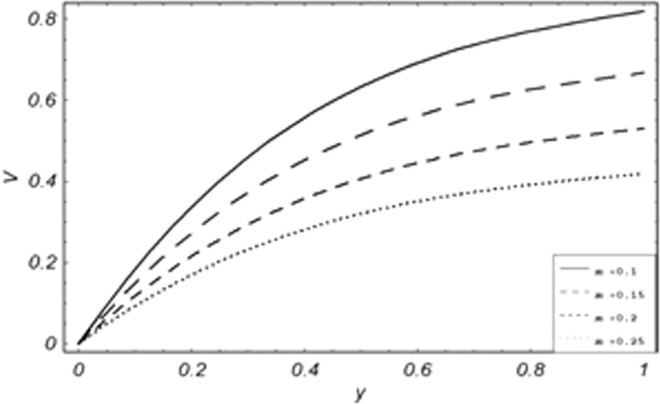
Variation of particle velocity with different values of *m*.

**Figure 13:**
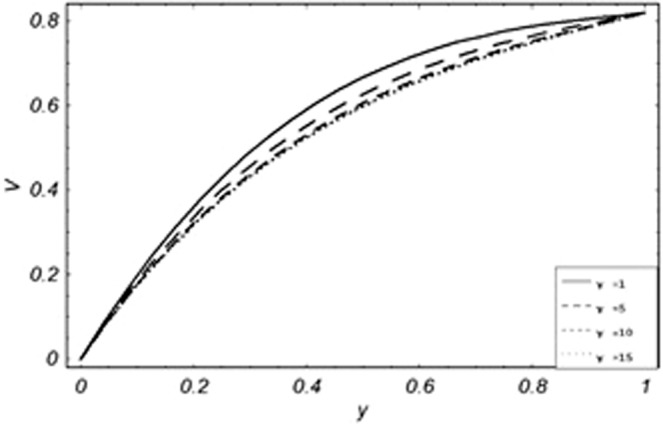
Variation of particle velocity with different values of *γ*.

**Figure 14:**
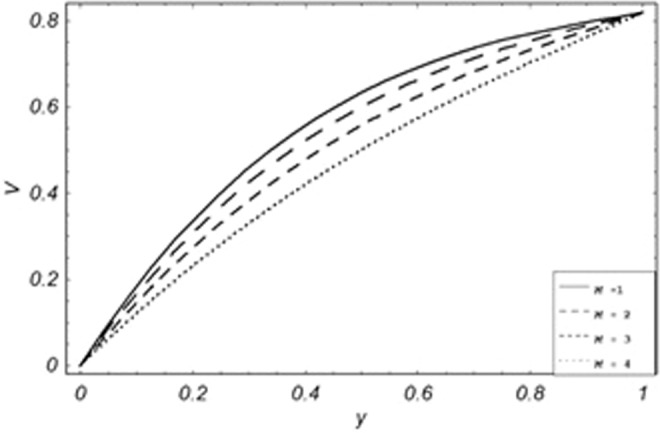
Variation of particle velocity with different values of *M*.

**Figure 15:**
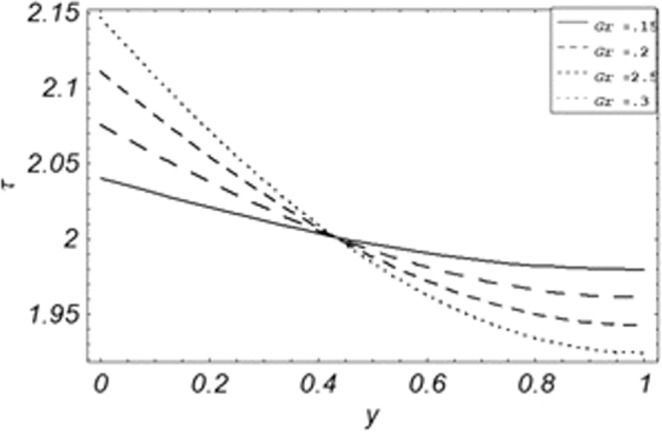
Evolution of shear stress with various values of *Gr*.

**Figure 16:**
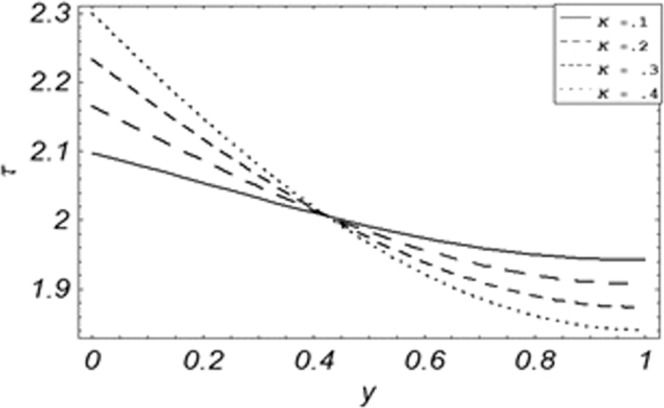
Evolution of shear stress with various values of *K*.

**Figure 17:**
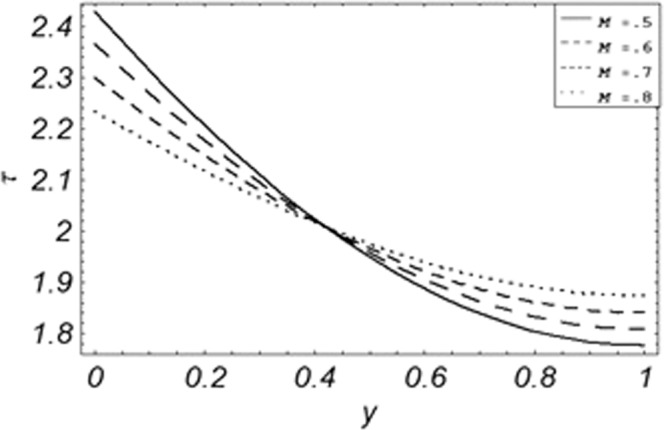
Evolution of shear stress with various values of *M*.

**Figure 18:**
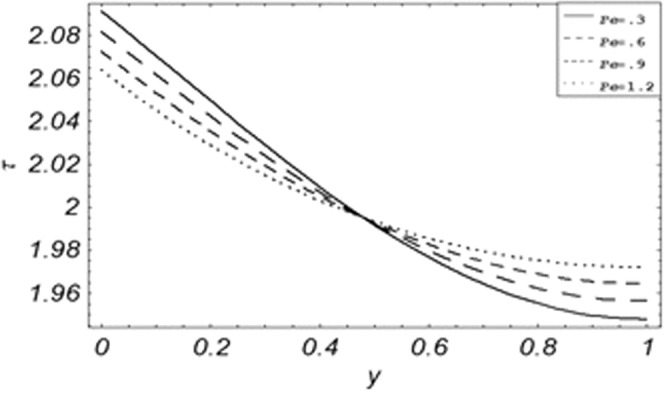
Evolution of shear stress with various values of *Pe*.

**Figure 19:**
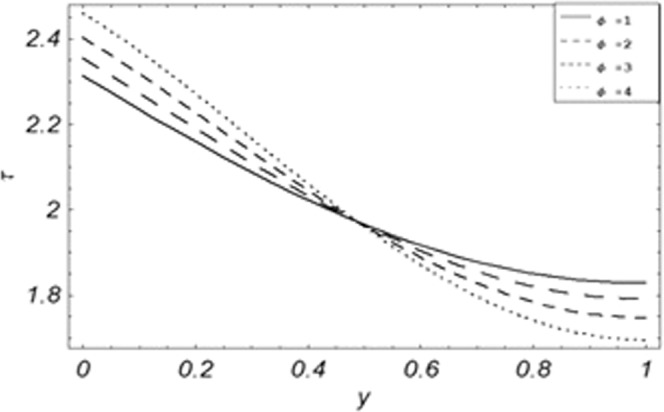
Evolution of shear stress with various values of *ϕ*.

**Figure 20:**
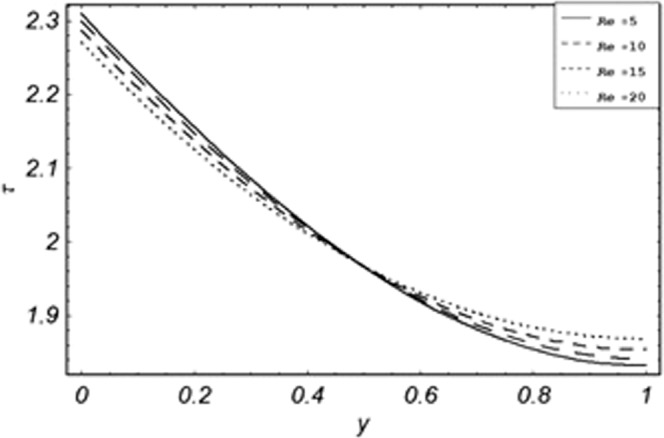
Evolution of shear stress with various values of *Re*.

*Gr*,*ϕ* and *K* accelerates the boundary layer velocity of the viscoelastic dusty fluid, shown from Figs. 2, 3 and 7. As *Gr* represent the ratio of bouncy to viscous forces, increase of *Gr* bring an increase in bouncy forces which cause to accelerate the boundary layer velocity, the coefficient of heat absorption *ϕ* have the same behavior with boundary layer velocity like *Gr*, increasing the numerical values of *ϕ* the fluid absorb more heat which decreases the viscous forces as a result increase occurs in fluid velocity, while increasing the dusty parameter *K*, increase occurs in the boundary layer velocity of the fluid. Figures 4–6 representing the decreasing behavior of boundary layer velocity with different values of *R*, *Pe* and *M* respectively. The concentration of the dust particle *R* is inversely related to the velocity of the fluid because the increase in concentration of the dust particles leads the dusty-phase to extra collisions, which increases the internal resistive forces, that’s why the increasing values of *R* decrease the boundary layer velocity of the fluid. As *Pe* represent the ratio of viscous to thermal forces, due to the dominant nature of viscous forces, the increment to *Pe* increase viscous forces which de-accelerates the boundary layer velocity of the fluid. The magnetic parameter *M* affects the internal resistive forces against the fluid flow called Lorentz forces, that’s why even in viscoelastic dusty fluids *M* can be used for boundary layer control.

From Figs. 8 to 14 only *Gr* shows the increasing behavior of the velocity of the dust particle while the increasing values of *R*, *Pe*, *m*, *γ*, *ϕ* and *M* the decreasing behavior of the particle’s velocity have been observed. The bouncy forces have a direct relation with the momentum of dust particles, because it reduces the resistive forces to the flow, so an increase of *Gr* increases the dust particle velocity shown from Fig. 9. Figure 10 explains the physical behavior of the velocity of the dust particle with increasing values *R*. When *R* increases it leads the internal motion of the dust particles to extra collisions, which causes retardation in the velocity of the dust particles. As discussed before that increase of *Pe* increases the dominancy of viscous forces over thermal forces, this increase of viscous forces is responsible to decrease the velocity of the dust particles depicted in Fig. 11. By Newton’s law of motion, mass is inversely related to displacement, same is the case in Fig. 12, the increasing values of the mass of dust particles retardation occur in the velocity of the dust particles. Time relaxation parameter, heat absorption, and magnetic parameter decrease particle velocity shown from Figs. 8, 13 and 14 respectively. As the dust particles are conducting, by absorbing heat the rate of collisions between adjacent particles increase which creates an opposing force to the flow of dust particles, similarly, the Lorentz forces also act against the motion of dust particle and a result decrease occurs in the velocity of the dust particles.

The evolution of applied shear stresses with different values of *Gr*, *K*, *M*, *Pe*, *ϕ* and Re have been pictured in Figs. 15–20 respectively. With the increasing values of *M*, *Pe* and Re applied shear stresses increasing while with the increase in *Gr*, *K* and *ϕ* applied shear stresses are decreasing. As previously discussed the relation of the magnetic parameter, Peclet number and Reynolds number with a fluid velocity, which shows retardation in fluid velocity, so this retardation is an agreement for an increase in internal resistive forces, that’s why shear stresses increasing with increase of aforementioned parameters, presented in Figs. 17, 18 and 20. Due to the fact of the dominant nature of viscous to thermal forces the internal resistive forces increase. Similarly, by increasing *M* velocity decreased. The physics behind this phenomenon is that, by increasing *M* the Lorentz forces increases which increases the internal resistive forces, and this increase of resistive forces retard the fluid velocity. That’s why a larger amount of applied shear stresses will be required to drive the fluid flow as shown in Figs. 15 and 17. The increase of bouncy forces and dusty parameter decreases the internal viscous (resistive) forces due to which smaller amount of applied shear stresses will be required to drive the fluid, that’s why shear stresses are decreases with increase in *Gr* and *K* shown in Figs. 15 and 16. The effect of heat absorption on the frictional forces of the fluid is shown in Fig. 19, which shows dual behavior. Above boundary layer the frictional forces increases while inside boundary layer the frictional forces decreases. When the fluid absorbs energy it accelerates the dust particles, this acceleration of dust particles enhances the fluid flow. So this enhancement in flow means that shear stresses are decreased. In the literature, about the relation of Reynolds number with skin friction one can find the increasing behavior of skin friction with increasing values of Re, same is the case with the current study as well, Re can be used for boundary layer control because it retards the velocity and increases the skin friction as clear from Fig. 20.

The numerical interpretation of the rate of heat transfer with various physical parameters of our interest has been shown in Table 1, The increasing values of *Pe* and *R* enhance the rate of heat transfer, while this rate of heat transfer retards with the increase in *γ*and *ϕ*. Similarly, Table 2, shows the effect of skin friction with different values of various physical parameters. The findings of this work conclude that the increasing values of *Gr*, *K*, *R*, Re, *γ* and *ϕ* enhances skin friction, while the increasing values of *Pe*, *M* and *α* the skin friction decreases.

## Conclusion

The theoretical investigation of the influence of numerous physical parameters on the unsteady MHD flow of viscoelastic dusty fluid flowing in the horizontal channel has carried out in this article. It is assumed that the flow is free stream fluctuating, incompressible, unidirectional and one-dimensional, electrically conducting and heat-absorbing, the heat transfer with free convection mood has also taken into account. The embedded dust particles are assumed to be conducting and homogenously distributed in the second-grade fluid. The concluding points of the current study are:

- The parametric influence of physical parameters on fluid velocity profile, shear stress, particle velocity profile, and temperature has discussed in detail. It is found that the increasing values of *Gr* increasing the velocities of both the fluid and dust phases.
- The heat absorption coefficient *ϕ* enhances the fluid velocity, also reduces the skin friction furthermore it retards the velocity of the particle.
- Unlike *Gr* the concentration of the dust particles *R* reduces the velocities of both phases. I.e. fluid phase and dusty phase.
- The increment in the mass of the dust particles decreases the velocity of the particle, because of Newton’s law of motion, acceleration is inversely related to mass of the object. That’s why increase in mass of the dust retards the velocity of the particle.

## Nomenclature

*u* → Boundary layer velocity of the fluid

*v* → Velocity of the dust particle

*U* → Free stream velocity

*T* → Temperature of the fluid

*T*_*p*_ → Temperature of the particle

*ρ* → Fluid density

*υ* → kinematic viscosity

*α*_1_ → Second grade parameter

*K*_0_ → Stock’s resistance coefficient

*N*_0_ → Number density of the dust particle which is assumed to be constant

*σ* → Electrical conductivity

*B*_0_ → Applied magnetic field

*g* → Gravitational acceleration

*β*_*T*_ → Coefficient of thermal expansion

*k* → Thermal conductivity of the base fluid

*c*_*p*_ → Specific heat capacity

*ρ*_*p*_ → Density of the dust particle

*γ*_*T*_ → Temperature relaxation time

*T*_*w*_ → Temperature of wall

*T*_∞_ → Ambient temperature

*c*_*s*_ → Specific heat capacity of the dust particle

*m* → Average mass of the dust particle

*M* → Non-dimensional parameter

*P*_*e*_ → Peclet number

*G*_*r*_ → Grashof number

Re → Reynolds number

*R* → Particle concentration parameter

*ϕ* → Heat absorption coefficient

*K*_1_ → Dusty fluid parameter

*K*_2_ → Dusty fluid parameter

*γ* → Non-dimensional temperature relaxation time parameter

*α* → Non-dimensional second grade parameter
